# Comparative changes of health-promoting phytochemicals and sugar metabolism of two hardy kiwifruit (*Actinidia arguta*) cultivars during fruit development and maturity

**DOI:** 10.3389/fpls.2022.1087452

**Published:** 2022-12-15

**Authors:** Yuanxiu Lin, Honglan Tang, Bing Zhao, Diya Lei, Xuan Zhou, Wantian Yao, Jinming Fan, Yunting Zhang, Qing Chen, Yan Wang, Mengyao Li, Wen He, Ya Luo, Xiaorong Wang, Haoru Tang, Yong Zhang

**Affiliations:** ^1^ College of Horticulture, Sichuan Agricultural University, Chengdu, China; ^2^ Institute of Pomology and Olericulture, Sichuan Agricultural University, Chengdu, China; ^3^ General Manager's Office, Sichuan Innofresh Agricultural Science and Technology Co., Ltd., Ya’an, China

**Keywords:** *actinidia arguta*, antioxidant capacity, nutritional compounds, organic acids, sugars

## Abstract

**Introduction:**

Hardy kiwifruit (*Actinidia arguta*) has an extensive range of nutritional and bioactive compounds and has been valued as a great resource for kiwifruit breeding. A better understanding of the dynamic changes of the composition and accumulation of nutritional compounds during fruit development and ripening is required before genetic or cultural improvements can be targeted.

**Methods:**

In the present study, the phytochemical analysis of two *A. arguta* cultivars ‘Yilv’ and ‘Lvmi-1’ showed that they comprised different morphology, with a higher fruit diameter while a lower vertical fruit diameter of ‘Lvmi-1’ compared with ‘Yilv’. The antioxidant capacity of both cultivars decreased during the maturity time and showed no significant difference between them. Furthermore, although glucose gradually increased during the maturity time, the predominant sugar composition was speculated to be fructose in ‘Lvmi-1’ fruit while sucrose in ‘Yilv’ fruit at the early fruit developmental stages. Moreover, the predominant acids in ‘Yilv’ and ‘Lvmi-1’ were citric acid followed by quinic acid, malic acid, and oxalic acid. The expression of sugar- and starch-related genes encoding the crucial enzymes suggested different changes in ‘Yilv’ and ‘Lvmi-1’. Notably, a subsequent correlation analysis showed a significant positive correlation between sucrose phosphate synthase (SPS) expression and glucose in ‘Yilv’, fructokinase (FK) expression, and starch content in ‘Lvmi-1’, implying their vital roles in sugar and starch accumulation. By contrast, a significant negative correlation between FK expression and fructose in ‘Lvmi-1’ fruit was observed.

**Results and Discussion:**

In summary, our results provide supplementary information for the dynamic changes of nutritional compounds and antioxidant capacity during hardy kiwifruit maturity time and give a clue for exploring the mechanism of sugar and starch accumulation in hardy kiwifruit.

## Introduction

Kiwifruit (*Actinidia*, Actinidiaceae), a worldwide commercially domesticated and cultivated perennial fruit tree, is a sought-after fresh fruit in the marketplace due to its delicious taste and multiple health-promoting nutritional components (Wang et al., 2021). Although there are 15 out of over 50 *Actinidia* species producing edible fruits, only three of them are now commercially available (Silva et al., 2019). Among them, *A. chinensis* and *A. deliciosa* have been suggested as the most commercially significant species (Follett et al., 2019), whereas increasing evidence has appeared to indicate that *A. arguta*, also known as hardy kiwifruit, kiwiberry, baby kiwi, or grape kiwi has a lot of horticultural advantages over kiwifruit, such as high frost hardiness (down to -30°C in the dormant period), richer bioactive compounds, stronger antioxidant activities, and edible skin (Latocha et al., 2015; Latocha, 2017; Wojdyło et al., 2017; Zhang et al., 2021).

Hardy kiwifruit (*A. arguta*) grew up first outside Asia but is native from northeast Asian countries. It is worldwide consumed and appreciated mainly due to its high phenolic, ascorbic acid (AsA, also known as vitamin C), and mineral contents (Latocha et al., 2015; Wojdyło and Nowicka, 2019). Moreover, hardy kiwifruit has been recognized as one of the most nutritionally rich fruits and an excellent source of health-promoting phytochemicals, such as sugars, organic acids, minerals, dietary fiber, vitamins (especially vitamin C), and antioxidants (mainly polyphenols) (Pinto et al., 2020). In addition, it has been shown that the antioxidative activity of hardy kiwifruit is strongly correlated with the content of AsA and polyphenols (Latocha et al., 2015). The much higher polyphenols and AsA in hardy kiwifruit compared with common kiwifruit (Leontowicz et al., 2016) has made hardy kiwifruit as a promising natural antioxidant. Due to these attractive health-promoting traits, hardy kiwifruit is of great interest to researchers, being a potential candidate to be included in a healthy and sustainable diet, and becoming popular to consumers.

In recent years, an increasing number of studies have been reported with subjects on the nutritional value and health benefits in hardy kiwifruit, most of which point to and confirm a rich composition of health-promoting ingredients, such as AsA, phenolics, anthocyanins, and sugars (Latocha, 2017; Wojdyło et al., 2017). However, even though the metabolite levels in hardy kiwifruit are generally higher than those in common kiwifruit, they vary in different hardy kiwifruit cultivars. Nowadays, there are various varieties of hardy kiwifruit on the market, the most representative and well-known are ‘Ananasnaya’, ‘Geneva’, ‘Weiki’, ‘Issai’, ‘Jumbo’, ‘Ken’s Red’, and ‘Maki’ (Pinto et al., 2020). It was found that the cultivar ‘Ananasnaya’ contains a higher AsA content compared with ‘Geneva’ (Latocha et al., 2013), whereas the AsA content found in one of the hardy kiwifruits *A. kolomikta* is over 1,000 mg/100 g of fresh weight (FW) (Latocha et al., 2010). The total sugar content ranges from 6.2 to 9.6 g/100 g FW in different hardy kiwifruit cultivars (Wojdyło et al., 2017). Cultivar ‘HR’ and ‘CJ-1’ comprise around two times higher total phenolic content than ‘LD-121’ (Zhang et al., 2021).

According to the authors, changes in biochemical, phenolic, and antioxidant properties in fruit considerably differ not only among cultivar types and growing regions but also in the degree of fruit maturity at harvest (Lee et al., 2015; Park et al., 2022). However, as far as we know, most recent studies focus on comparing the phytochemical and antioxidant properties of selected fruit cultivars at commercial harvest, but there is little information on the changes that occur in nutritional composition of hardy kiwifruit during maturation (Boldingh et al., 2000; Han et al., 2019; Li et al., 2021). In order to better understand the nutritional value offered by hardy kiwifruit during the maturity stages of the fruit, it is imperative to study the changes in the nutritional contents of commercially grown hardy kiwifruit cultivars. The objective of this study was to quantify the accumulation of nutritional compounds and antioxidant capacity in two hardy kiwifruit cultivars ‘Yilv’ and ‘Lvmi-1’ grown in Sichuan Province, China, at different fruit maturity stages. We first determined the dynamic phenotypic changes of these two cultivars during fruit maturity. Subsequently, the composition of sugars and organic acids was assessed to give more detailed information about the physiochemical changes. Finally, the antioxidant capacity was also measured during fruit maturity. These findings would be beneficial for providing critical information on selecting the best harvest stage of these varieties, which would facilitate the extension of cultivation of these two cultivars and the improvement of other kiwifruit varieties.

## Materials and methods

### Plant materials and sample preparation

The fruit samples were the same with those in our previous study (Lin et al., 2022). Fruits of ‘Yilv’ and ‘Lvmi-1’ were collected from Sichuan Innofresh Agricultural Science and Technology Co., Ltd. A total of about 1,000 flowers from at least 150 plants (4 years old) were tagged. Fruits at 30 days post anthesis (DPA), 40 DPA, 50 DPA, 60 DPA, 70 DPA, and 80 DPA were collected for measurement. For each cultivar, 90 fruits were harvested without physical injuries in total, 60 of which were used for biological trait determination in three biological replicates with 20 fruits each. The other 30 fruits were frozen in liquid nitrogen and stored at −80°C and were used for RNA extraction and real-time quantitative PCR (qPCR) analysis. Each 10 fruits were randomly selected as one biological replicate.

### Measurement of fruit phenotypic characteristics

Fruit length and width referring to the vertical diameter and fruit diameter, respectively, were measured using a vernier caliper. The fruit firmness was measured using a penetrometer (model SMTT50; Toyo Baldwin, Tokyo, Japan) fitted with a 5-mm plunger.

### Determination of titratable acidity and total soluble solids

Titratable acidity (TA) was determined by titration aliquots of homogenate of fresh fruits by 0.1 N NaOH to an end point of pH 8.1 using an automatic pH titration system (pH meter type IQ 150; Warsaw, Polska) and expressed as citric acid equivalents. Total soluble solids (SSC) were determined in fresh juices with a refractometer (Atago RX-5000, Atago Co., Ltd., Japan) and expressed as °Brix.

### Quantification of starch and sugar content

Soluble sugars, including glucose, fructose, and sucrose, were extracted and analyzed as previously described with some modification (Li et al., 2019). Fresh samples (100 mg) were added to 5 ml of distilled water and homogenized for 1 min. The mixture was then extracted in a water bath at 80°C for 30 min. The supernatant was collected after centrifugation at 8,000g at room temperature for 5 min, filtered through a 0.45-μm cellulose acetate filter, and then analyzed by HPLC using an Agilent 1260 instrument equipped with a refractive index detector (Agilent Technologies, Inc., Palo Alto, USA). Samples (10 μl) were separated at 35°C on an Agilent ZORBAX carbohydrate column (250 × 4.6 mm i.d.; 5 μm particle size) using 80% acetonitrile at a flow rate of 1.0 ml per min. Contents of individual soluble sugars (glucose, fructose, and sucrose) were determined using the standard calibration curves for each sugar (Sangon Biotech Co., Ltd., Shanghai, China).

Starch content was measured as previously described (Li et al., 2021). The precipitation obtained when extracting sugars was autoclaved for 1 h and then incubated in 55°C with amyloglucosidase in 0.25 M acetate buffer (pH 4.5) for 1 h. The sample was then centrifuged for 10 min at 7,000 rpm, and the supernatant was used to determine the starch concentration using a colorimetric measurement.

### Extraction, identification, and quantification of organic acid compounds

Organic acid compound extraction was conducted according to the previous method (Wojdyło et al., 2017), with slight modifications. Fresh sampled fruits (0.5 g) were mixed with 20 ml of H_2_O, boiled at 100°C for 20 min, sonicated for 15 min, and centrifuged at 10,000 g for 15 min. The supernatant was filtered through a 0.45-μm cellulose acetate filter and then analyzed by HPLC using an Agilent 1260 instrument equipped with a refractive index detector (Agilent Technologies, Inc., Palo Alto, USA). The elution system consisted of 40 mM KH_2_PO4–H_3_PO4 buffer (pH 2.4) with a flow rate of 0.6 ml min^−1^. Organic acids were separated on an ODS C18 column (250 × 4.6 mm i.d.; 5 μm particle size) and detected using a diode-array detector set up at 210 nm. Standard curves for pure standards of organic acids (oxalic, citric, malic, and quinic acids) (Sigma, Poole, Dorset, UK) were used for quantification.

### Determination of total phenolic content and total flavonoid content

Total phenolic content (TPC) of the hardy kiwifruit was measured using the Folin–Ciocalteu method with minor modification (Lee et al., 2019). The fruit extract (300 μl) was mixed with Folin–Ciocalteu’s phenol reagent (400 μl); after 5 min in darkness, 2 ml of 5% Na_2_CO_3_ was added, and the final volume was adjusted to 10 ml with distilled water. After incubating for 60 min at room temperature, the absorbance value was read at 765 nm using a spectrophotometer. TPC was expressed in mg gallic acid equivalents (GAE)/100 g FW.

Total flavonoid content (TFC) was measured using a modified colorimetric method described by Kim (Kim et al., 2003). The fruit extract (150 μl) was mixed with deionized water (3.2 ml) and 5% (w/v) NaNO_2_ solution (150 μl) and incubated at room temperature for 5 min. A volume of 150 ml of 10% (w/v) Al(NO_3_)_3_ solution was added to the mixture and further incubated at room temperature for 1 min. The mixture was mixed with 1 M NaOH (1 ml) and measured immediately at 510 nm using the spectrophotometer. TFC of hardy kiwifruit was expressed in mg rutin equivalents (RE)/100 g FW.

### Antioxidant capacity assays

2,2-Azino-bis(3-ethylbenzothia-zoline-6-sulfonic acid) (ABTS), 2,2-diphenyl-1-picrylhydrazyl radical (DPPH), and ferric reducing antioxidant power (FRAP) assays were conducted by the previous method (Barreca et al., 2011). For ABTS assay, 5 ml aqueous ABTS solution (7 mM) was added to 88 µl of 140 mM of a potassium per sulfate solution, which was heated in a water bath at 70°C for 30 min to create ABTS radicals. The solution of ABTS radicals was adjusted to an absorbance of around 0.650 at 734 nm. The reaction between the ABTS radical solution (980 ml) and the appropriately diluted extracts (20 ml) was conducted at 37°C for 10 min. Absorbance at 734 nm was measured using the spectrophotometer immediately. DPPH was determined as described. The absorbance of fresh DPPH radicals in 80% (v/v) aqueous methanol was set to 0.650 at 517 nm. The reaction between DPPH radical solution (2.95 ml) and the appropriately diluted extracts (50 ml) took place at 23°C for 30 min. The reduction of absorbance at 517 nm was measured immediately. FRAP reagent was prepared by mixing 200 ml acetate buffer (300 mM, pH 3.60), 20 ml TPTZ solution (10 mM in 40 mM HCl), and 20 ml of FeCl_3_·6H_2_O solution (20 mM). FRAP reagent (3.80 ml) was added to 200 µl of fruit extract. After 30 min at ambient temperature, the absorbance was detected at a wavelength of 593 nm. The results of ABTS, DPPH, and FRAP were represented as µmol of Trolox equivalent (TE) per gram dry wight (µmol TE/g DW) for fruits.

### Real-time qPCR reactions

Total RNA was isolated by using the improved CTAB method (Lin et al., 2022) and subsequently quantified by electrophoresis and using a NanoDrop nucleic acid protein analyzer. First-strand cDNA was synthesized referring to the instructions of the reverse transcription kit of Beijing Quanshijin Biotechnology Co., Ltd. qPCR reactions were carried out with SYBR qPCR Mix using a Bio-Rad CFX96 Real-Time System. *AaActin* was used as the internal control to normalize gene expression. The relative expression levels of genes were calculated based on the 2^−ΔΔCT^ method. The primer sequences were designed using Primer Premier 5.0 software, and the sequence information is listed in **Table S1** (**Supplementary Table 1**). The expression of six key enzymes involved in sugar and starch metabolism were detected, including neutral invertase (NI), soluble acid invertase (SAI), sucrose synthase (SS), sucrose phosphate synthase (SPS), hexokinase (HK), and fructokinase (FK).

### Data analysis

Statistical analysis was performed using SPSS 19.0 software (SPSS Inc., Chicago, IL, USA), and significant differences among the samples were calculated using one-way ANOVA followed by Duncan’s multiple-range test at the 5% level (*p* < 0.05). Data were expressed as the means ± SD (standard deviation), and each sample has three independent repeats. Principal component analysis (PCA) and Pearson correlation matrix were performed using Origin 2017 and the statistical program R (version 3.5.0), respectively. Correlation analysis was also conducted by SPSS 19.0 software using Pearson’s method.

## Results

### Morphological characteristics of fruit

**Figure 1:**
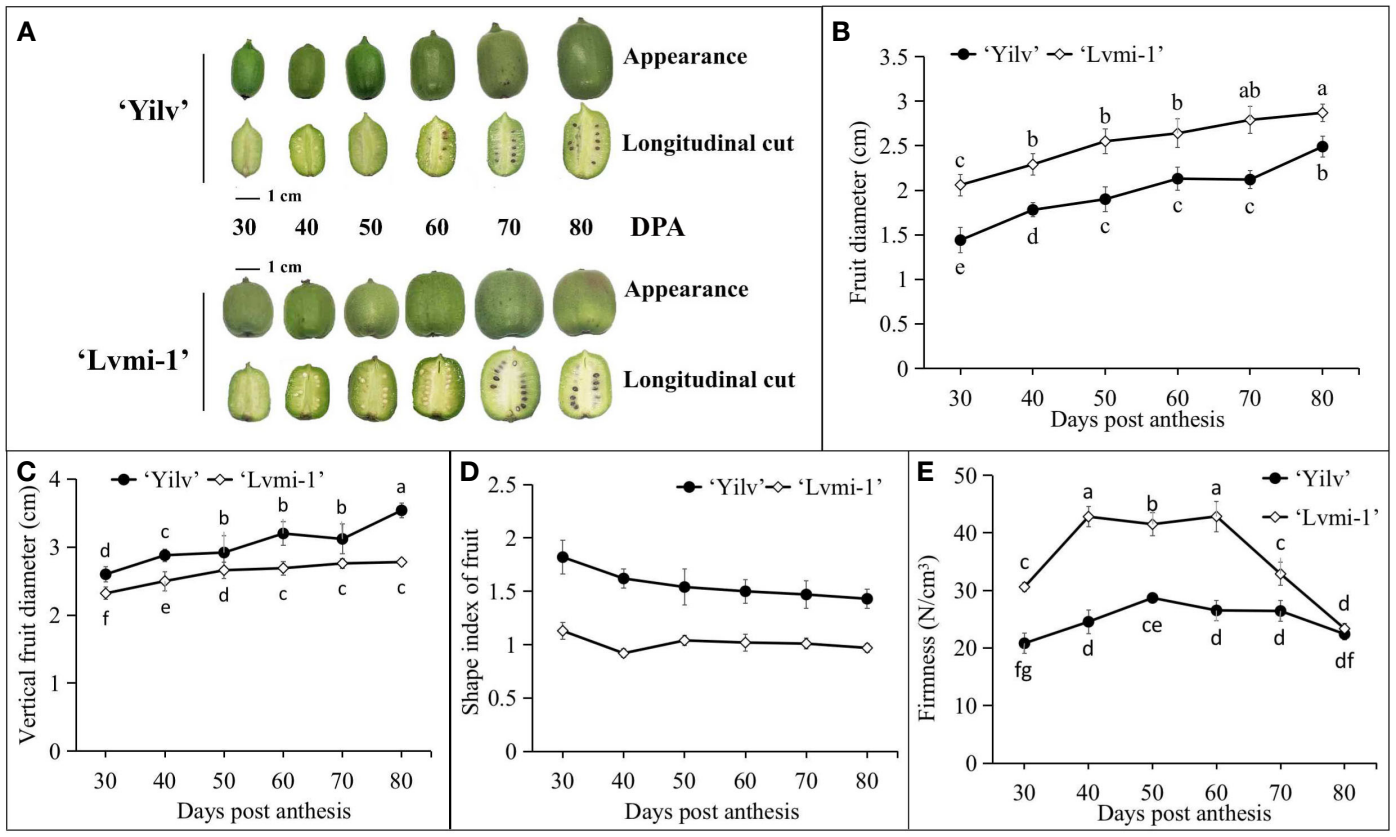
Morphological characteristics of the ‘Yilv’ and ‘Lvmi-1’ fruits. **(A)** Fruit phenotypes during development and maturity. **(B)** Diameter of fruit changes during fruit development. **(C)** Changes of vertical diameter of fruit in different developmental stages. **(D)** Shape index of fruit during development. **(E)** Firmness changes in different developmental stages of ‘Lvmi-1’ and ‘Yilv’ fruit. Data were represented by the mean values ± standard deviation. Different letters indicated significant difference at the *p* ≤ 0.05 level.

As shown in **Figure 1A**, a visual inspection of six different representative stages during fruit development and maturity of ‘Yilv’ and ‘Lvmi-1’ appeared with different morphology, which was supported by the following equatorial diameter (**Figure 1B**), vertical fruit diameter (**Figure 1C**), and shape index of fruit (**Figure 1D**) measurement during the development. The results (**Figure 1**) showed that ‘Yilv’ and ‘Lvmi-1’ are both in small size with fruit diameter below 3 cm at 80 DPA. Moreover, the fruit diameter of ‘Lvmi-1’ was significantly higher than that of ‘Yilv’, whereas the vertical fruit diameter of these two cultivars showed no significant difference. Thus, the shape index of fruit (ratio of vertical diameter and equatorial diameter) of ‘Lvmi-1’ was significantly lower than ‘Yilv’. Furthermore, the seeds of the ‘Yilv’ fruit appeared to turn to black at 60 DPA, whereas the seeds of the ‘Lvmi-1’ fruit turned to black around 10 days later at 70 DPA. This result of firmness determination showed that the firmness of the ‘Lvmi-1’ and ‘Yilv’ fruits decreased at 70 and 60 DPA, respectively, and ‘Lvmi-1’ comprised a significant higher firmness level than ‘Yilv’ at the early stages, whereas no significant difference of firmness in ‘Yilv’ and ‘Lvmi-1’ at the mature stage (80 DPA) was observed, indicating that the decrease of ‘Lvmi-1’ firmness was more rapid than that of ‘Yilv’ during development and maturity (**Figure 1E**). It was also found that there were two rapid growth periods (50–60 and 70–80 DPA) of ‘Yilv’ fruit, during which the fruit length and diameter increased rapidly, while the fruit of ‘Lvmi-1’ appeared a steady increase during the whole development process (**Figure 1**).

### Conventional nutrient content during fruit development

**Figure 2:**
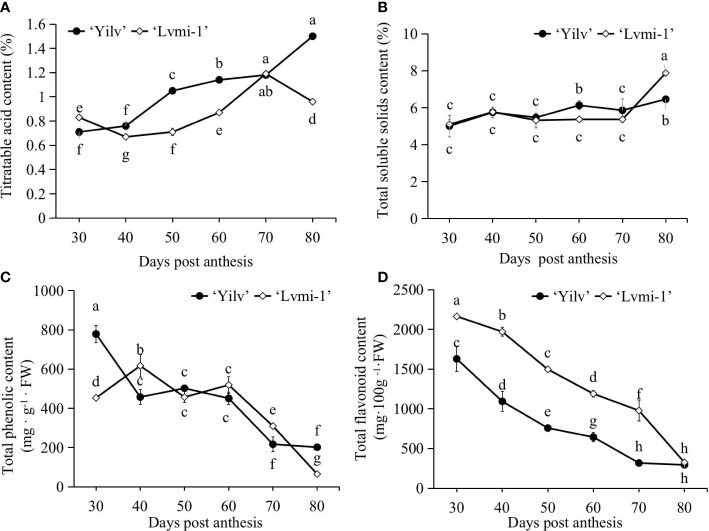
Changes of conventional nutrient contents in ‘Yilv’ and ‘Lvmi-1’ fruits during fruit development and maturity. **(A)** Titratable acid content changes during fruit development. **(B)** Total soluble solid content changes during fruit development. **(C)** Total phenolic content changes during fruit development. **(D)** Total flavonoid content changes during fruit development. Data were represented by the mean values ± standard deviation. Different letters indicated significant difference at *p* ≤ 0.05 level. The titratable acid content was expressed as citric acid equivalents.

According to the results, a general increase of TA (**Figure 2A**) and SSC (**Figure 2B**) in ‘Yilv’ and ‘Lvmi-1’ was observed. A significant higher TA level while a lower SSC level was shown in ‘Yilv’ than ‘Lvmi-1’ at the mature stage (80 DPA) (**Figure 2B**). No significant difference of SSC between ‘Yilv’ and ‘Lvmi-1’ was observed during the fruit development and maturity (30–70 DPA). In addition, the antioxidants including total phenolic and flavonoid were also estimated. As a result, the TPC and TFC showed a gradual decrease during fruit development and maturity (**Figures 2C, D**). There was no significant difference of TPC in ‘Yilv’ and ‘Lvmi-1’ during fruit development, except for that at 30 and 80 DPA, in which ‘Yilv’ exhibited a comparatively higher level of TPC than ‘Lvmi-1’. By contrast, the fruit of ‘Yilv’ contained a lower level of TFC than the ‘Lvmi-1’ fruit during the fruit development until 80 DPA.

### Antioxidative characterization during fruit development

**Figure 3:**
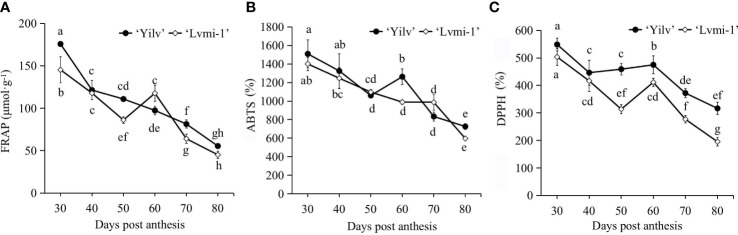
Changes of antioxidant activity of ‘Yilv’ and ‘Lvmi-1’ fruits during fruit development and maturity. **(A)** Ferric reducing antioxidant power, FRAP. **(B)** 2,2’-Azino-bis (2-ethylbenzothizoline-6-sulfonic acid), ABTS. **(C)** 2,2-Diphenyl-1-picrylhydrazyl radical, DPPH. Data were represented by the mean values ± standard deviation. Different letters indicated significant difference at the *p* ≤ 0.05 level.

Since the fruit maturity is recognized as an oxidative process, the antioxidative activity during the fruit development and maturity was measured to characterize the differences between two cultivars. Generally, as detected by FRAP (**Figure 3A**), ABTS (**Figure 3B**), and DPPH scavenging activity (**Figure 3C**), the antioxidative activity gradually decreased during fruit development and ripening. There was no significant difference between ‘Yilv’ and ‘Lvmi-1’ with ABTS level. For the FRAP level, a significant difference was only observed at 30, 50, and 60 DPA. However, except for the level at 30 and 40 DPA, the DPPH level was higher in ‘Yilv’ fruit than in ‘Lvmi-1’. Moreover, the highest antioxidative activity was detected by ABTS (**Figure 3B**), followed by DPPH (**Figure 3C**), and the lowest was detected by FRAP (**Figure 3A**), but the general change pattern during fruit development detected by these three different methods was the same.

### Changes of organic acids in ‘Lvmi-1’ and ‘Yilv’

**Figure 4:**
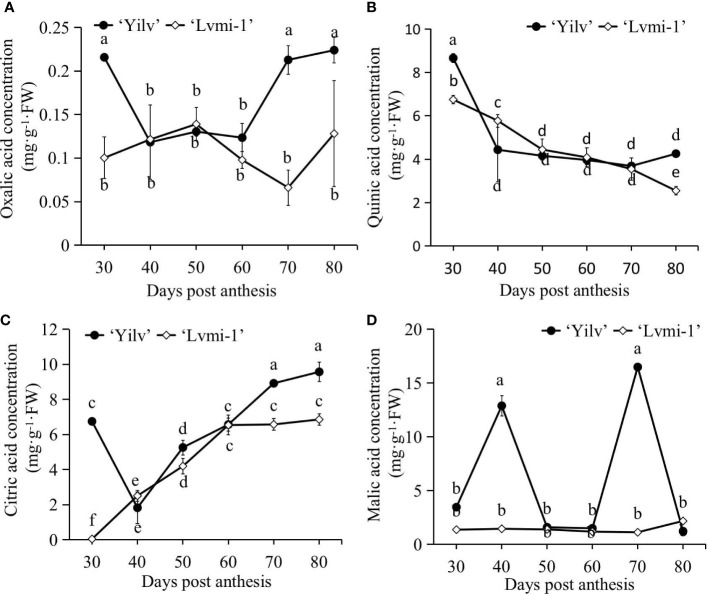
Changes of organic acid composition and content of ‘Yilv’ and ‘Lvmi-1’ fruits during fruit development and maturity. **(A)** Oxalic acid content. **(B)** Quinic acid content. **(C)** Citric acid content. **(D)** Malic acid content. FW, fresh weight. Data were represented by the mean values ± standard deviation. Different letters indicated significant difference at the *p* ≤ 0.05 level.

The accumulation of organic acid is one of the most important traits of fruit development and maturity; to elucidate more differences between hardy kiwifruit cultivar ‘Yilv’ and ‘Lvmi-1’ during the fruit ripening, the dynamic changes of organic acid components were determined by HPLC methods. Based on the results, oxalic acid (**Figure 4A**) showed no obvious increase or decrease patterns in both cultivars. It remained at a relatively stable level during the development of ‘Lvmi-1’. The content of quinic acid (**Figure 4B**) gradually increased during fruit development, and a higher level in ‘Yilv’ at 30 and 80 DPA was observed compared with ‘Lvmi-1’, whereas citric acid (**Figure 4C**) gradually increased from 30 to 80 DPA in both cultivars and a higher level in ‘Yilv’ fruit was found. Furthermore, malic acid (**Figure 4D**) did not show a regular change pattern in both cultivars, and it did not show significant change during fruit development of ‘Lvmi-1’. In addition, the oxalic acid content was the lowest compared with other three acids, the highest content proved to be quinic acid at the early developmental stages while citric acid at the late maturity stages in both ‘Yilv’ and ‘Lvmi-1’.

### Changes of starch and sugar during the fruit development

**Figure 5:**
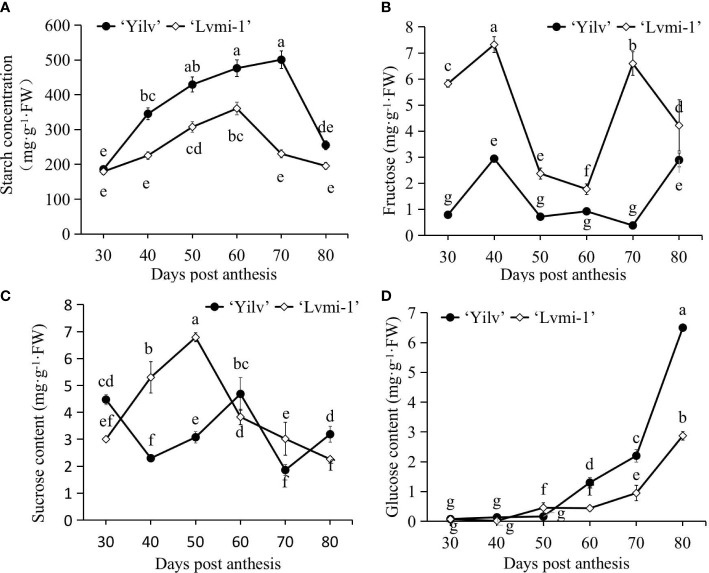
Changes in sugar and starch content of ‘Yilv’ and ‘Lvmi-1’ fruit during fruit development and maturity. **(A)** Starch content. **(B)** Fructose content. **(C)** Sucrose content. **(D)** Glucose content. FW, fresh weight. Data were represented by the mean values ± standard deviation. Different letters indicated significant difference at the *p* ≤ 0.05 level.

As shown in **Figure 5A**, it was evident that the starch level increased from 30 to 60 and 70 DPA, respectively, in ‘Yilv’ and ‘Lvmi-1’ fruits, and it was sharply decreased thereafter until 80 DPA. A significantly higher level of starch was observed in ‘Yilv’ from 40 to 70 DPA compared with ‘Lvmi-1’. In terms of sugar content, the fructose content accumulated at a high level at 30 and 40 DPA; thereafter, it decreased until 60 DPA and increased again at 70 and 80 DPA (**Figure 5B**). The sucrose content increased and reached a peak at 50 DPA and decreased during the later maturity stages in ‘Lvmi-1’, whereas it decreased from 30 to 40 DPA and then increased to 60 DPA and finally decreased at 70 DPA and increased at 80 DAP (**Figure 5C**). Although the glucose content showed a gradual increase during the whole fruit development and maturity, the accumulation level of glucose was the lowest compared with the fructose and sucrose content in the ‘Lvmi-1’ fruit (**Figure 5D**). Similar change patterns were also observed in ‘Yilv’ fruit (**Figure 5**). Interestingly, the predominant sugar was speculated to be fructose in the ‘Lvmi-1’ fruit while sucrose in the ‘Yilv’ fruit according to the highest level of the three determined sugar components.

### Principal component analysis and correlation analysis

**Figure 6:**
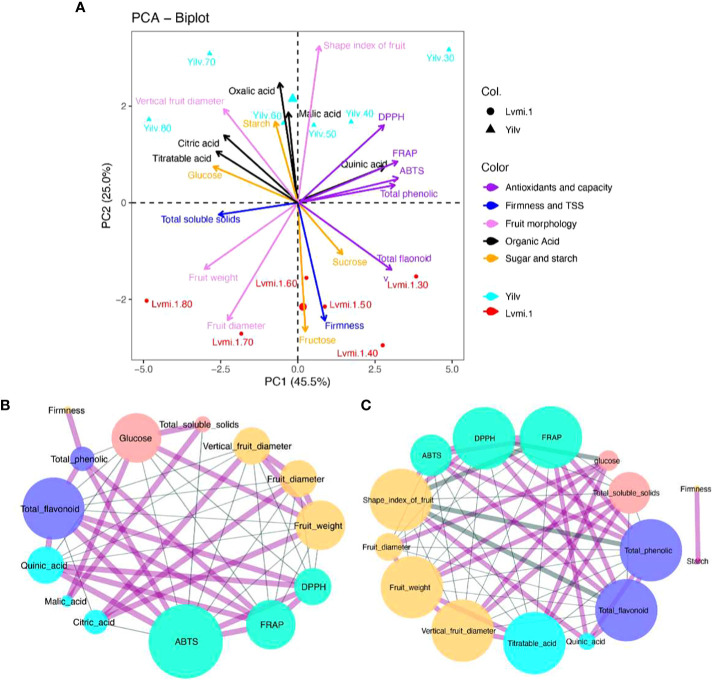
PCA biplot and correlation analysis. **(A)** PCA biplot. **(B)** Correlation analysis in ‘Lvmi-1’. **(C)** Correlation analysis in ‘Yilv’. Correlation pairs with |r|>0.8 are shown; the edge indicates coefficient r values. The red line indicates a positive correlation; the blue line indicates a negative correlation. Different colors of node circles present different types of compounds: purple indicates the antioxidants, yellow represents fruit morphological characteristics, pink and blue indicate sugar and organic acids, respectively, and the green circles indicate the antioxidant capacity. Size of nodes indicates the correlated node numbers.

In order to identify the necessary components explaining the greater part of variances, all of the physiological changes in the two cultivars were subjected to PCA. As a result (**Figure 6A**), the first and second principal components (PC1 and PC2) accounted for 45.5% and 25.0% of the total variation, respectively. The points of different developmental stages were obviously separated along PC1, whereas the points of different cultivars were mainly separated along PC2, revealing the variance between cultivars and developmental stages. In addition, the content of quinic acid, total phenolic, and antioxidant capacity was positively related to the early (DPA 30–60) developmental stages of ‘Yilv’. Meanwhile, the organic acid content was positively related to the late stages in ‘Yilv’. By contrast, in ‘Lvmi-1’, a performance of sugar (sucrose and fructose), total flavonoid, and firmness was observed in the early developmental stages, and a preference of fruit weight, diameter, and TSS in the late stages of ‘Lvmi-1’ occurred.

Correlation analysis suggested that FRAP, ABTS, and DPPH were positively correlated in both ‘Yilv’ and ‘Lvmi-1’ cultivars (**Figures 6B, C**), implying the consistency and accuracy of antioxidant capacity using different detection methods. Moreover, TPC and TFC were significantly and positively correlated with each other, indicating their similar change trend. Specifically, in the ‘Lvmi-1’ fruit, only TFC was significantly positively correlated with FRAP, ABTS, and DPPH (**Figure 6B**), suggesting it might contribute more to the total antioxidant capacity than TPC in this cultivar. Furthermore, a strong negative correlation between fruit morphology and antioxidant capacity was observed, indicating a decrease trend for antioxidant capacity during fruit development. Citric acid and quinic acid were shown to be highly positively and negatively related to fruit morphology, respectively. However, in the ‘Yilv’ fruit, TPC and TFC showed a high positive correlation with FRAP, ABTS, and DPPH during development (**Figure 6C**), revealing their significant contribution on the antioxidant capacity. Glucose content and TSS were suggested to be positively correlated with fruit morphology. A strong correlation between starch and firmness was also found in ‘Yilv’ fruit.

### Expression analysis of the starch and sugar metabolism-related genes during fruit development

**Figure 7:**
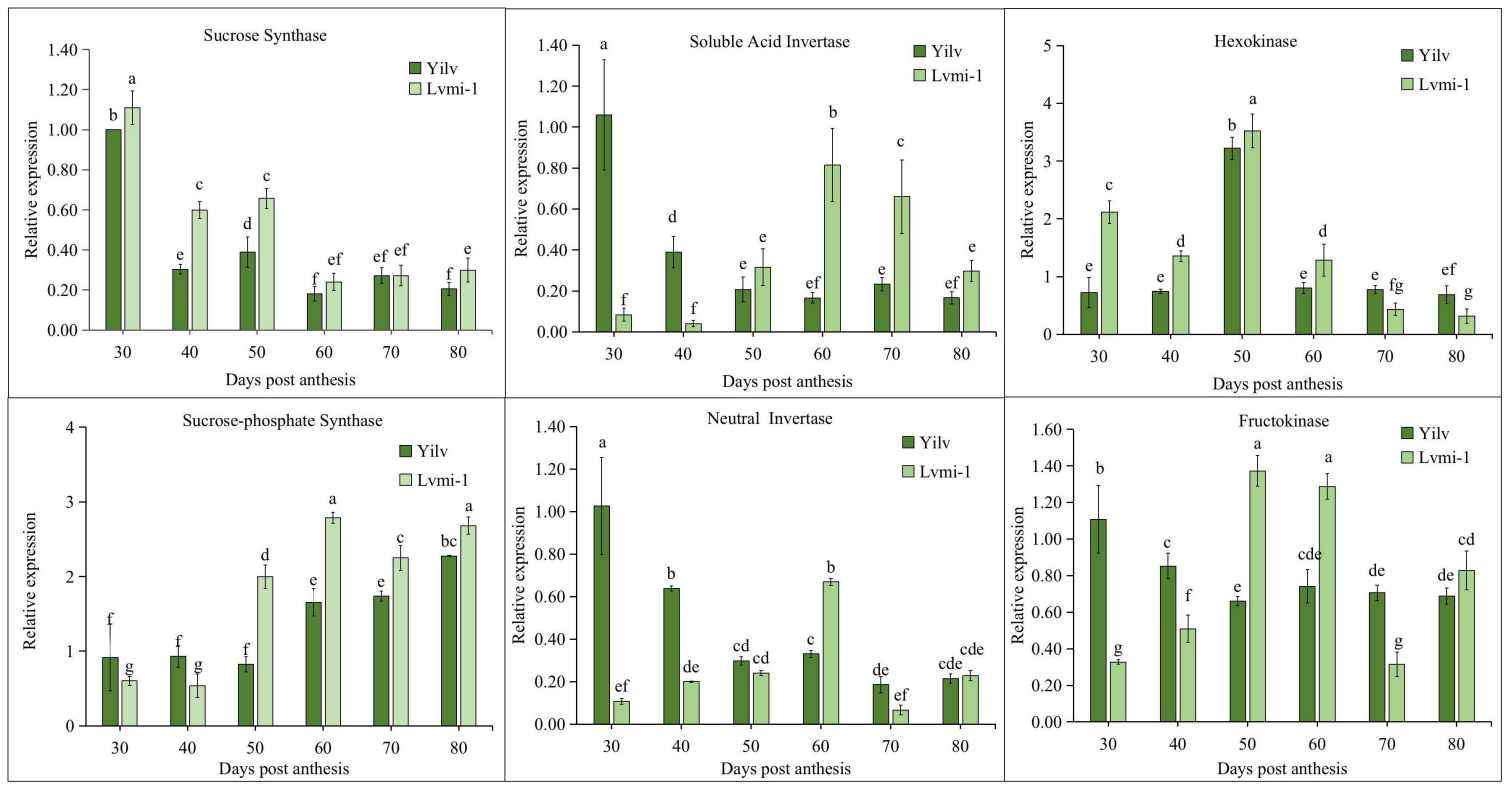
Expression profiling of genes related to sugar and starch metabolism during fruit development in ‘Yilv’ and ‘Lvmi-1’. Relative expression was normalized against the value in the ‘Yilv’ fruit at 30 DPA. Error bars indicated the standard deviation of three biological replicates. Different letters indicated significant difference at the *p* ≤ 0.05 level.

To deeply explore the molecular mechanism of starch and sugar changes during fruit development in these two cultivars, the expression of related key genes was detected by qPCR. It was shown (**Figure 7**) that the highest level of *SS* gene expression appeared at 30 DPA in both cultivars, then it gradually decreased from 40 to 80 DPA. A higher level of *SS* expression was observed in the early developmental stages (30 to 60 DPA), whereas no significant difference was found on 60, 70, and 80 DPA. On the contrary, the expression of the *SPS* gene displayed the opposite trend. The expression of *SPS* steadily increased from the early stage to the late mature stage, and a significantly higher level occurred in ‘Lvmi-1’ from 50 to 80 DPA. The expression of *SAI* and *NI* exhibited similar decrease change patterns in both cultivars. They both peaked at 30 DPA and gradually decreased with fruit maturity in ‘Yilv’, whereas they increased first from 30 to 60 DPA and decreased subsequently in ‘Lvmi-1’. The highest *HK* expression was detected at 50 DPA in both ‘Lvmi-1’ and ‘Yilv’, whereas the expression at other developmental stages was low and no obvious change was observed. For the expression of *FK*, it decreased from 30 to 50 DPA and thereafter remained at a relatively stable level in ‘Yilv’. In ‘Lvmi-1’, it increased from 30 to 50 DPA and showed a slight decrease then. These results indicated different expression patterns of the key related genes in two cultivars.

### Correlation analysis of the starch and sugar content and related gene expression

To investigate the relationship between gene expression and the content of sugar and starch, Pearson correlation analysis was conducted. As shown in **Table 1**, most of the gene expressions were negatively correlated with sugar and starch in the ‘Yilv’ fruit during development. An exception was observed in *HK* and *SPS* expressions, which exhibited a positive correlation with sucrose, fructose, and starch content, although only *SPS* expression and glucose were correlated at a significant level (**Table 1**). Interestingly, in the ‘Lvmi-1’ fruit, the gene expression of *FK* was significantly negatively correlated with fructose content but positively correlated with the content of starch. Similarly, *NI* expression also showed a highly positive correlation with starch, indicating they had important roles in the accumulation of starch during fruit development (**Table 2**). Moreover, the expression of *SS* was positively correlated with sucrose and fructose but negatively correlated with glucose and starch, whereas a totally opposite correlation was found in *SPS* and *SAI* expressions. The expressions of *HK* and *NI* displayed a positive correlation with sucrose and starch but a negative correlation with glucose and fructose. Overall, these results indicated the different accumulation of sugar and starch and corresponding potential molecular mechanism in ‘Yilv’ and ‘Lvmi-1’.

**Table 1:** Correlation coefficient between expression of related key genes with sugar and starch content in ‘Yilv’.

	Expression level						Sucrose	Glucose	Fructose	Starch
	SS	HK	NI	SPS	SAI	FK			
Sucrose	-0.099	0.801	-0.027	-0.728	-0.200	-0.240	1			
Glucose	-0.420	-0.178	-0.570	0.853*	-0.446	-0.485	-0.544	1		
Fructose	-0.314	-0.330	0.011	0.233	-0.170	-0.060	-0.082	0.418	1	
Starch	-0.658	0.282	-0.686	0.101	-0.702	-0.681	0.346	-0.184	-0.439	1

*Significant differences at the 0.05 level.

**Table 2:** Correlation coefficient between expression of related key genes with sugar and starch content in ‘Lvmi-1’.

	Expression level						Sucrose	Glucose	Fructose	Starch
	SS	HK	NI	SPS	SAI	FK			
Sucrose	0.203	0.790	0.102	-0.238	-0.195	0.508	1			
Glucose	-0.521	-0.561	-0.065	0.621	0.116	0.074	-0.544	1		
Fructose	0.262	-0.387	-0.745	-0.665	-0.502	-0.932**	-0.244	-0.142	1	
Starch	-0.422	0.334	0.819*	0.513	0.670	0.822*	0.494	-0.280	-0.769	1

*Significant differences at the 0.05 level.

**Significant differences at the 0.01 level.

## Discussion

### The general morphological characterization and nutritional content of hardy kiwifruit

In China, hardy kiwifruit is considered as an underutilized fruit; the varieties are only cultivated and consumed in certain provinces. The two cultivars in the present study are cultivated in Ya’an, Sichuan; little information about their physicochemical changes during maturity is available until now. Morphological appearances and physicochemical features including TSS, firmness, AsA content, and TA are fruit quality traits which could be used to indicate the maturation, harvest time, and shelf life (Wang et al., 2021). Fruit maturity is depicted by a decrease in firmness, TA reduction, and TSS increase (Krupa et al., 2011; Mitalo et al., 2019). Therefore, it could be speculated that ‘Lvmi-1’ starts to mature earlier at 60 DPA, whereas ‘Yilv’ might be later at 70 DPA according to our results obtained in this study. From 60 DPA, the ‘Lvmi-1’ fruit showed a slow increase in fruit size, a significant decrease in fruit firmness, a rapid increase in fructose content, and a peak of starch content, whereas these changes started from 70 DPA in the ‘Yilv’ fruit. Moreover, TSS and TA can reflect the taste of fruit. The TSS increased rapidly from 70 to 80 DPA (**Figure 2**), which were similar among the two cultivars. This was similar with other studies on TSS in *A. deliciosa*, *A. chinensis*, and *A. arguta* (Boldingh et al., 2000; Ainalidou et al., 2016; Li et al., 2021). Correspondingly, the TA started to decrease in ‘Lvmi-1’ at 70 DPA, indicating that the fruit of this cultivar might start to form a good taste. Overall, the seasonal fruit quality and physiological characteristics reported here could provide basic data for evaluating the harvest time of hardy kiwifruit cultivars ‘Yilv’ and ‘Lvmi-1’.

### The antioxidant capacity in hardy kiwifruit

The TPC and TFC were among the most important antioxidant compounds contributing to total antioxidant capacity. Various studies have revealed a positive correlation between the phenolic content and antioxidant activity (Wojdyło and Nowicka, 2019). Consistent with this, the correlation analysis in the present study showed that the TPC and TFC were significantly positively correlated with the antioxidant capacity in ‘Yilv’ fruit (**Figure 6C**), indicating their important contributions on antioxidant capacity. This is consistent with a previous study (Jeong et al., 2020), in which a high correlation between TPC, TFC, and antioxidant capacity during the cold storage of hardy kiwifruits was proposed, whereas in the ‘Lvmi-1’ fruit, only a correlation between TFC and antioxidant capacity was observed (**Figure 6B**), suggesting that their contribution may vary with cultivars in hardy kiwifruit. It has also been suggested that TPC and TFC are significantly different among the hardy kiwifruit genotypes at the maturity stage (Wang et al., 2018). Similarly, our results showed that ‘Yilv’ contained significantly higher TPC than the ‘Lvmi-1’ fruit (**Figure 2C**), indicating a probable higher antioxidant capacity of the ‘Yilv’ fruit than that of ‘Lvmi-1’. This was also supported by the subsequent antioxidant capacity assays, which showed a slightly higher level of antioxidant capacity (ABTS, DPPH, and FRAP) in ‘Yilv’ than in ‘Lvmi-1’ (**Figure 2**). Moreover, the antioxidant capacity determined by three different methods in this study have shown similar patterns during the maturity time, indicating the reliability of the determination.

### The organic acid, starch, and sugar changes in hardy kiwifruit

Generally, most hardy kiwifruits have citric and quinic acids as their predominant organic acids, followed by malic acid (Latocha, 2017; Wojdyło et al., 2017). Consistent with this, our results have found that the predominant acids in ‘Yilv’ and ‘Lvmi-1’ were citric acid followed by quinic acid, malic acid, and oxalic acid (**Figure 4**). The citric acid level gradually increased during the maturity time and was determined to be 5–10 g/100 g FW in the mature ‘Yilv’ and ‘Lvmi-1’ fruits (**Figure 4C**), which is consistent with a previous report (Wojdyło et al., 2017). However, the content of hardy kiwifruit was only 0.5–1.5 g/100 g FW in Japan (Nishiyama et al., 2008), which might be due to the different growth areas of plants. Moreover, quinic acid was prevalent in the young fruit and decreased thereafter (**Figure 4B**), whereas the amount of malic acid and oxalic acid was little affected by fruit maturity in ‘Lvmi-1’, which is the same with previous results (Kim et al., 2012). The two abnormal-like peaks of malic acid at 40 and 70 DPA in the ‘Yilv’ fruit might be caused by the measured sample specificity. The balance between sugars and organic acids strongly influences the sensory properties and fruit palatability (Jaeger et al., 2003). It has been suggested that the main soluble sugars in kiwifruit (*A. deliciosa*) are glucose and fructose, with a smaller amount of sucrose present, whereas sucrose is the predominant sugar in hardy kiwifruit (*A. arguta*) followed by glucose and fructose (Park et al., 2006; Kim et al., 2012). At the mature stage, the highest level of sugar was proven to be glucose in ‘Yilv’ and sucrose in ‘Lvmi-1’; our results (**Figure 5**) show similar results. The hardy kiwifruit also accumulates a high level of starch. The two cultivars detected in this study showed similar changes in starch content over the fruit development period. For ‘Yilv’, starch accumulation occurred between 40 and 70 DPA, which is longer than that of ‘Lvmi-1’ between 40 and 60 DPA (**Figure 5A**). This difference probably resulted from dilution caused by cultivar difference in growth (Moscatello et al., 2011); the larger growth of ‘Lvmi-1’ than ‘Yilv’ fruit might cause the decrease and continuous increase in starch content in corresponding cultivar. A decrease in starch level was observed for both cultivars (**Figure 5A**) in the late developmental stages, which is similar with other studies (Li et al., 2021). This decrease might be caused by the conversion of starch to total sugar content (Ghasemnezhad et al., 2013).

### Key genes involved in sugar metabolism in hardy kiwifruit

Sweetness is one of the most important quality traits for kiwifruit cultivation and breeding. Sugars including sucrose, fructose, and glucose are important molecules in plants and function in many processes (Smeekens et al., 2010). However, there was limited information about the expression analysis of key genes involved in sugar metabolism in hardy kiwifruit. Here, the expression analysis of key genes encoding the crucial enzymes related to sugar metabolism showed that *SS* was highly expressed in immature fruit and gradually decreased in subsequent stages of fruit development, whereas *SPS* exhibited a low expression in immature fruit and a higher expression level in mature stages (**Figure 7**). This result is in consistent with that of *A. chinensis* as previously suggested (Tang et al., 2016). Moreover, *SPS* expression significantly positively correlated with glucose content in the ‘Yilv’ fruit (**Table 1**), which indicated the important role of *SPS* in glucose accumulation. Fructose, another one of the most important sugars in kiwifruit, can be phosphorylated to fructose-6-phosphate by *FK* (Granot, 2007). It was suggested by our results that in the ‘Lvmi-1’ fruit, *FK* expression negatively correlated with fructose but positively correlated with starch (**Table 2**), implying the conversion from fructose to starch content.

## Conclusions

In summary, the phenotypes and metabolites vary with fruit development and ripening in cultivars of hardy kiwifruit. Specifically, the ‘Yilv’ fruit contains higher TFC and starch and glucose content than ‘Lvmi-1’, whereas the ‘Lvmi-1’ fruit might start to mature around 10 days earlier than the ‘Yilv’ fruit under the growth condition in Ya’an, Sichuan. Moreover, *SPS* and *FK* could be developed as candidates to regulate the sugar and starch content in hardy kiwifruit. The findings reported here contribute to a better understanding of the changes of nutritional compounds and antioxidants during fruit development and maturity in hardy kiwifruit.

## Data availability statement

The original contributions presented in the study are included in the article/**Supplementary Material**. Further inquiries can be directed to the corresponding authors.

## Author contributions

Authors’ contributions: Conceived and designed the experiments: HT and YZ. Performed the experiments: YXL, HLT, BZ, XZ. Analyzed the data: YXL, DL and WY. Wrote the paper: YXL. Revised the paper: JF, YTZ, QC, YW, ML, YL, WH and XW. All authors contributed to the article and approved the submitted version.
